# Introducing an efficient model for the prediction of placenta accreta spectrum using the MCP regression approach based on sonography indexes: how efficient is sonography in diagnosing accreta?

**DOI:** 10.1186/s12884-020-2799-0

**Published:** 2020-02-17

**Authors:** Mahboobeh Boroomand fard, Maryam Kasraeian, Homeira Vafaei, Mojgan Akbarzadeh Jahromi, Payam Arasteh, Hadi Raeisi Shahraki, Peyman Arasteh

**Affiliations:** 1 Maternal-Fetal Medicine Research Center, Shiraz University of Medical Sciences, Shiraz, Iran; 2 Shiraz Breast Diseases Research Center, Shiraz University of Medical Sciences, Shiraz, Iran; 3 Department of Biostatistics and Epidemiology, Faculty of Health, Shahrekord University of Medical Sciences, Shahrekord, Iran

**Keywords:** Morbidly adherent placenta, Placenta accreta spectrum, Ultrasonography, Accreta, Diagnosis

## Abstract

**Background:**

For the first time, we aimed to introduce a model for prediction of placenta accreta spectrum (PAS), using existing sonography indices.

**Methods:**

Women with a history of Cesarean sections were included. Participants were categorized “high risk” for PAS if the placenta was previa or low-lying.

Sonography indices including abnormal placental lacuna, loss of clear zone, bladder wall interruption, myometrial thinning, placental bulging, exophytic mass, utero-vesical hypervascularity, subplacental hypervascularity, existence of bridging vessels, and lacunar flow, were registered.

To investigate simultaneous effects of 15 variables on PAS, Minimax Concave Penalty (MCP) was used.

**Results:**

Among 259 participants, 74 (28.5%) were high risk and 43 individuals had PASs.

All sonography indices were higher among patient with PAS (*p* < 0.001) in the high risk group.

Our model showed that utero-vesical hypervascularity, bladder interruption and new lacunae have significant contribution in PAS. Optimal cut off point was *p* = 0.51 in ROC analysis. Probability of PAS for women with lacunae was between 96 and 100% and probability of PAS for women without lacunae was between 0 to 7%, therefore accuracy of the proposed model was equal to 100%.

**Conclusions:**

Using the introduced model based on three factors of abnormal lacuna structures (grades 2 and 3), bladder wall interruption and utero-vesical vascularity, 100% of all cases of PASs are diagnosable. If supported by future studies our model eliminates the need for other imaging assessments for diagnosis of invasive placentation among high risk women with previous history of Cesarean sections.

## Background

Invasive placentation or placenta accreta spectrum (PAS) refers to a spectrum of disorders in which the placenta attaches in a pathological manner to the myometrium [1]. In developed countries PAS remains to be the most common cause for Cesarean hysterectomy [2], on the other hand the most important risk factor for increased PAS rates are increased Cesarean sections [2]. The incidence of the condition has been estimated to have increased from 1 in every 2500 pregnancies since the 1990s [3] to one in every 500 to 600 pregnancies [4]. According to the degree of trophoblastic invasion through the myometrium three variants can be recognized for PAS, which include 75% as PAS, 18% as increta, and 7% as percreta [3].

Placenta accreta (used to define all three conditions) is associated with multiple complications. Among these complications include: internal organ damage, hemorrhage, respiratory distress, thromboembolic events, infections, coagulopathies, genitourinary complications and finally multi-organ failure and death [5, 6].

Prenatal diagnosis of placenta accreta has been shown to decrease mortality and morbidity associated with the condition, as it allows planned intervention [7]. Diagnosis of the condition is done through imaging modalities including MRI and ultrasonography (US), however definitive diagnosis of the condition is based on pathological evaluation after hysterectomy [8, 9]. US remains to be the first line diagnostic modality among patients suspected of placenta accreta due to factors like easiness of use, easy access, the minor invasive nature and the lower expense compared to MRI [9].

Multiple indices have been introduced in US which are used for the diagnosis of accreta, however the same signs have shown varying accuracy for the diagnosis of accreta in different studies. This has been attributed to factors like the subjective nature and operator dependency of sonography, different study design and eventual diagnosis of the accreta [10, 11].

Recently, the European Working Group on Abnormally Invasive Placenta (EW-AIP) released a report on existing sonography parameters for the diagnosis of accreta in order to improve international comparisons and to unify definition of accreta in literature and to provide standard definitions for US indices [12].

To date, multiple studies have compared sonography and MRI for the diagnosis of accreta [8, 10]. Moreover, some studies have also focused on each sonography index and have separately defined accuracy of each sonography index for the diagnosis of accreta [9, 13, 14]. These studies lack homogeneity and differ in their reports, mainly due to the lack of a unified definition.

In this study, using the definitions provided by the EW-AIP, we aimed to define a standard model for the prediction of PAS among women with a history of Cesarean section, using existing sonography indices. We further compared each index regarding their agreement and prediction power for PAS based on the gold standard diagnostic modality, separately.

## Methods

### Study setting and patient selection

This is a prospective cross-sectional study performed in Hafez Hospital affiliated to Shiraz University of Medical Sciences, Shiraz, Iran. In this study all women with a history of at least one previous Cesarean section, during March 20th 2016 to February 19th 2017, were considered for inclusion in the study. Among these, all women in their second and third trimester of pregnancy who referred to the perinatology ward of the medical care center for the evaluation of site of placenta and placental adhesion, were included in the study. All women who referred during their first trimester were asked to refer during their second trimester as well.

**Fig. 1:**
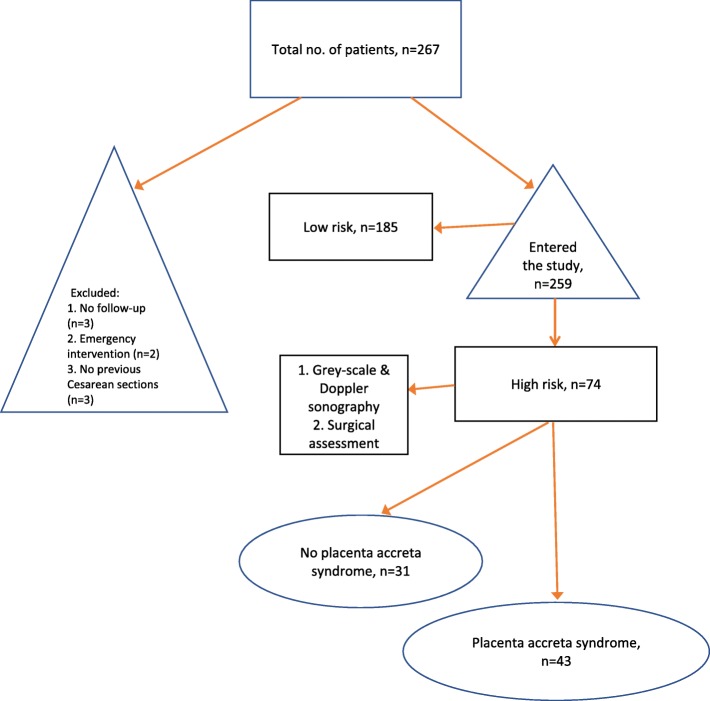
Flow diagram showing patient recruitment

Individuals without a history of Cesarean sections, and those who did not refer for their follow-ups were excluded from the study. Figure 1 shows the flow diagram related to patients’ recruitment.

### Study protocol

Every individual underwent systematic two-dimensional grey-scale US imaging (GE Voluson R 730, GE medical systems, Zipf, Austria). Initially, site of placenta was evaluated using abdominal transducers. In cases of suspicion of low lying placenta or placenta previa, trans-vaginal sonography was done, during which patients had an empty bladder and the distance between placental edge and the internal cervical orifice was measured. The term “placenta previa” was used when the placental edge overlaped or was within 2 cm of the internal cervical orifice in late pregnancy, and the term “low-lying” was used if the placental edge was located further than 2 cm but within 3.5 cm from the internal cervical orifice. In these cases, trans-vaginal US were repeated, during which the bladder was filled with 300 ml of liquid for better evaluation of the uterine serosa-bladder interfaces. Accordingly, participants were categorized in the “high risk” group for PAS if the placenta was previa or low-lying.

For placentas that contained abnormal vessels or lacunae structures, color Doppler sonography was done in order to map the vascularization of the intraplacental and uterine serosa-bladder interface and to measure the velocity flow of the inter-lacunae turbulant.

Patients classified as high risk for PAS were further re-evaluated just before planned delivery.

In each of the US and Doppler examinations the following parameters were examined: 1) presence of multiple placental lacunae. These irregularly shaped hypoechoic vascular spaces larger than 1*1 cm in size within the placental parenchyma, may give the placenta a “Swiss cheese” appearance, often containing turbulent flow that is visible on gray scale imaging. According to Finberg’s grading scale for lacunae [15], the structures were categorized as followed: grade 1+ for one to three lacunae structures, which are usually small, grade 2+ for four to six lacunae structures, which are typically larger, and grade 3+ which shows diffuse lacunae throughout the placenta, 2) losing or irregularity in the echolucent area between the placenta and the myometrium (clear space), 3) thinning or interruption of the hyperechoic interface between the uterine serosa and bladder wall (bladder line), 4) presence of an exophytic mass caused by intrusion of the placental tissue into the bladder, 5) sub-placental hypervascularity, 6) utero-vesical vascularity, 7) bridging vessels that extended from the placenta into the bladder wall via the myometrium. In case of existence of feeding vessels into the lacunae, velocity of blood flow within the lacunae structures was also measured.

Sonography imaging was done mainly during the third trimester, except in women who presented with history of rupture of membranes or vaginal bleeding or any emergency condition that would place that individual at risk of termination of pregnancy, during which US was done at any gestational age. In cases with multiple US examinations, the last scan before delivery was considered for study.

All known and suspected cases of PAS were admitted to the high risk pregnancy department at 33–34 weeks of gestation and a formal multidisciplinary management and treatment program was initiated for them by a trained team [16].

### Surgical assessment

According to our protocol, surgery was done with a surgical oncology team, general surgery team, and a urology team stationed at the high risk center, in which blood products were easily accessible and facilities such as postoperative ICU care was available. Moreover, a neonatal care department and a neonatal team were also present at the center. Surgery was initiated with adequate hydration and among patients who were suspected of adhesion, according to preoperative sonography, a fundal incision was made. With a fundal hysterotomy the child was delivered. The umbilical cord was closed at the nearest site to the placenta. If the patient was hemodynamically stable, without any effort to separate the placenta, a quick evaluation was done in 1–2 min. If the placenta had no invasion to the outer wall of the uterus and there was no bulging or bridging vessels existing between the uterus and the intestines or the bladder, moreover the patient remained hemodynamically stable, if the placenta had completely separated during this time, a standard typical Cesarean section would be performed. If the placenta was separated from all regions and was only attached in a few centimeters from the middle, if the surgery team would determine that by ligation of vessels or even removal of a few centimeters of the uterus, they are able to separate the placenta and save the uterus, then this approach was done. In cases in which during after birth, the patient was hemodynamically unstable, hemodynamic resuscitation was initiated and hysterectomy was done. Moreover, in cases where, the patient was hemodynamically stable, however the placenta had invaded the outer wall of the uterus or in cases where dilated or bridging vessels were seen between the uterus and the bladder or intestines, after birth and clamping of umbilical vessels, hysterectomy was done.

There was no attempt to remove the placenta manually.

For the purpose of this study, invasive placentation was suspected based on clinical assessment of abnormal adherence and evidence of gross placental invasion at time of surgery, after which histopathological study of the excised uterus confirmed the diagnosis of PAS.

All participant had declined conservative treatment (preserving the uterus with the placenta left in situ) or didn’t fulfill criteria for this treatment approach.

### Pathology evaluation

The uterus and the attached placenta were first weighed and measured. The external surface of the uterus, especially the anterior and lower segments, was evaluated for any hematoma, ruptured and bulging areas. Areas suspicious of percreta were inked and then the uterus was bivalved using a knife. The specimens were fixed in 10% neutral-buffered formalin overnight. Serial bread-loaf sections of the uterus were checked for areas of increta/percreta. Multiple sections were taken from suspicious areas of accreta and myometrial invasion. Moreover, two sections from the cervix or lower uterine segment (in supra-cervical hysterectomy) were taken to represent placenta previa. Four full wall thickness sections of non-attached areas of placental disc and sections of membrane and umbilical cord were taken for evaluation of placental abnormality. All sections were stained with hematoxylin and eosin and were then evaluated by an expert pathologist.

The absence of decidua between the placental villi and myometrium was considered placenta accreta, deeper invasion into the myometrium was considered increta, and complete invasion through the uterine was considered percreta [17].

### Definition of variables

Location of placenta in sonography was classified as: anterior high, fundal region posterior high, lateral high, anterior low lying, posterior low lying, lateral low, anterior previa, and posterior previa.

Data on age, number of Cesarean sections, gestational age at which diagnosis of PAS was considered, gestational age of delivery, gestational age of first Cesarean section, risk factors including: previous rupture of uterus, dilation and curettage, other operations, myomectomy, and sonography indices including abnormal placental lacuna, loss of clear zone, bladder wall interruption, myometrial thinning, placental bulging, exophytic mass, utero-vesical hypervascularity, subplacental hypervascularity, existence of bridging vessels, lacunar flow, and final diagnosis were registered for each patient.

High pressure in placental lacuna was considered as blood velocity higher than 15 cm/s [18].

In order to increase coherency and objectivity of study, all definitions of sonography indices used in the study, were done according to the unified definitions proposed by the EW-AIP 2016 [12]. Moreover, to remove any inter-observer bias, all sonography evaluations were done by a single radiologist who was blinded to the classification of patients (high risk or low risk groups).

In our study we considered grade 2 and grade 3 lacuna according to the the EW-AIP description as “new lacuna”.

### Statistical analysis

All univariate analysis was done using the SPSS® software for windows®, version 18, (SPSS Inc., Chicago, IL, USA). Initially individuals were classified as high risk and low risk groups (based on the existence of low lying placenta or placenta previa), after which they were categorized as PAS and non-PAS (based on pathology reports) and compared accordingly. For comparison of qualitative data the Chi-square test and the Fisher’s exact test and for comparison of normally distributed quantitative data, the independent-test was used. Furthermore, for comparison of quantitative data without a normal distribution the Mann-Whitney test was used.

The Kappa test was used to evaluate the overlap and agreement of each sonography index with the gold standard modality, for the diagnosis of PAS.

The receiver operator characteristic (ROC) curve analysis was used to define sensitivity and specificity of each sonography index compared to the gold standard diagnostic modality for the diagnosis of PAS. A cut-off point was also defined based on lacunar flow among individuals with lacunar structures to estimate the occurrence of PAS. The Youden index was used to define the optimal cut-off point based on lacunar flow for diagnosing PAS. The Youden index considers a point on the ROC curve optimum which has the maximum sensitivity and specificity [19].

Due to the nature of PAS rates among pregnant individuals, and due to the relatively small sample size and large number of variables, using traditional logistic regression was not appropriate in this setting. To investigate simultaneous effects of 15 variables on PAS, Minimax Concave Penalty (MCP) was used. Like other penalized models, degree of shrinkage in MCP is regulated by the tuning parameter which is a positive constant and estimates using the cross validation technique. Classification accuracy of the proposed model was investigated by obtained probability of PAS for each case and optimal cut-off point was determined using the ROC curve analysis. Statistical analysis was performed using SPSS 18.0 and ncvreg package in R 3.3.1 software.

In the agreement analysis and the regression model abnormal lacuna structures were re-defined into a different variable as new lacuna. New lacuna was categorized into two categories as followed: those with grade 0 and 1 were considered as no lacuna and those with grades 2 and 3 as positive for new lacuna.

A *p*-value of less than 0.05 was considered statistically significant.

## Results

During the study period, a total of 3 patients did not have a history of Cesarean sections and were referred due to thinning of the myometrium and 3 patients did not refer for their follow-up visits. In total, 2 individuals had emergency interventions before 28 weeks. This included women who presented with vaginal bleeding or premature rupture of membrane or any emergency condition that would place that individual at risk of termination of pregnancy. These individuals were excluded from the study.

Table one shows baseline and clinical characteristics in both high risk and low risk groups. A total of 259 individuals entered the study, among which 74 (28.5%) patients were classified as high risk. From the total 74 high risk patients, 31 of them had spontaneous separation of placenta in the operating room and only underwent Cesarean section. A total of 43 individuals in this group, had Cesarean hysterectomy due to a high suspicion of PAS in the operating room, which was further confirmed with pathology evaluation.

Individuals in the high risk group were older (31.43 ± 4.48 vs. 33.19 ± 4.25; *p* = 0.004), had a lower number of Cesarean sections (1.61 ± 0.77 vs. 1.93 ± 0.76; *p* = 0.003), a lower gestational age of diagnosis (32.01 ± 5.35 vs. 34.80 ± 3.46; *p* < 0.001), and a lower gestational age at time of delivery (34.14 ± 4.97 vs. 38.30 ± 1.87; *p* < 0.001) (Table 1).

**Table 1:** Baseline and clinical characteristics of the study population*

Variable	Sub group	Low risk (*n* = 185)	High risk (*n* = 74)†	*p*-value
Age - yrs		31.43 ± 4.48	33.19 ± 4.25	0.004
Site of placenta - no. (%)	Anterior high	142 (76.3)	0	< 0.001
	Fundal posterior high	34 (18.3)	0	
	Lateral high	10 (5.4)	0	
	Anterior low lying	0	9 (12.2)	
	Posterior low lying	0	7 (9.5)	
	Lateral low lying	0	2 (2.7)	
	Anterior previa	0	18 (24.3)	
	Posterior previa	0	6 (8.1)	
Number of Caesarean sections - median (interquartile range)		2 (1, 2)	1 (1, 2)	0.003
Gestational age at diagnosis - wks		34.80 ± 3.46	32.01 ± 5.35	< 0.001
Gestational age at time of delivery - wks		38.30 ± 1.87	34.14 ± 4.97	< 0.001
Time of first Caesarean section - wks		4.90 ± 2.70	4.96 ± 2.87	0.88
Risk factors - no. (%)‡	No	171 (92.4)	67 (90.5)	0.614
	Yes	14 (7.6)	7 (9.5)	

*All plus-minus values are means and standard deviations unless stated otherwise

†High risk individuals were those who had low lying placenta or placenta previa

‡Risk factors included: rupture of uterus, dilation and curettage, myomectomy and other operations

Among individuals who had PAS, higher rates of abnormal placental lacuna was detected (p < 0.001), furthermore higher lacunar flow was also detected (16.59 ± 8.63 vs. 1.02 ± 3.69, p < 0.001).

All other sonography indices evaluated among high risk patients, as expected, were higher among patient with PAS compared to those without PAS (Table 2).

**Table 2:** Sonography related indices among individuals classified as high risk*

Variable	Sub group	PAS (*n* = 43)	No PAS (*n* = 31)	*P*-value
Abnormal placental lacuna	No	0	170 (78.7)	< 0.001
	Grade 1	4 (9.3)	28 (13)	
	Grade 2	12 (27.9)	17 (7.9)	
	Grade 3	27 (62.8)	1 (0.5)	
Lacunar flow		16.59 ± 8.63	1.02 ± 3.69	< 0.001
Bladder interruption	No	6 (14)	31 (100)	< 0.001
	Yes	37 (86)	0	
Myometrial thinning	No	15 (34.9)	30 (96.8)	< 0.001
	Yes	28 (65.1)	1 (3.2)	
Placental bulging	No	25 (58.1)	31 (100)	< 0.001
	Yes	18 (41.9)	0	
Exophytic mass	No	25 (58.1)	31 (100)	< 0.001
	Yes	18 (41.9)	0	
Vesicular hypervascularity	No	0	28 (90.3)	< 0.001
	Yes	42 (100)	3 (9.7)	
Subplacental hypervascularity	No	5 (11.6)	29 (93.5)	< 0.001
	Yes	38 (88.4)	2 (6.5)	
Bridging vessels	No	4 (9.3)	31 (100)	< 0.001
	Yes	39 (90.7)	0	
Loss of clear zone	No	20 (46.5)	30 (96.8)	< 0.001
	Yes	23 (53.5)	1 (3.2)	

PAS: placenta accreta spectrum

*All plus-minus values are means and standard deviations, unless stated otherwise. Sonography indices were only evaluated in patients who were considered high risk (except for evaluation of lacunar structures and subsequently, lacunar blood flow)

When comparing each sonography index, existence of utero-vesical hypervascularity (kappa: 0.916, sensitivity: 90.3%, specificity: 100%), bridging vessels (kappa: 0.891, sensitivity: 100%, specificity: 90.7%), bladder interruption (kappa: 0.838, sensitivity: 86%, specificity: 100%), and subplacental hypervascularity (kappa: 0.808, sensitivity: 85.3%, specificity: 95%) showed the highest agreement for the diagnosis of PAS when cross compared to the gold standard diagnosis (Table 3).

**Table 3:** Agreement between sonography indexes and gold standard measurement for diagnosis of placenta accreta spectrum*

Variables	Accuracy	Kappa	Sensitivity	Specificity	*P*-value
Utero-vesical hypervascularity	95.9 (88.6–99.1)	0.92	90.3 (74.3–98)	100 (91.8–100)	< 0.001
Bridging vessels	94.6 (86.7–98.5)	0.89	100 (88.8–100)	90.7 (77.9–97.4)	< 0.001
Bladder interruption	91.9 (83.2–97.0)	0.84	86.0 (72.1–94.7)	100 (88.8–100)	< 0.001
Subplacental hypervascularity	90.5 (81.5–96.1)	0.81	85.3 (68.9–95.1)	95.0 (83.1–99.4)	< 0.001
New lacuna†	91.5 (87.4–94.6)	0.73	98.0 (95.0–99.5)	68.4 (54.8–80.1)	< 0.001
Myometrial thinning	78.4 (67.3–87.1)	0.58	96.8 (83.3–99.9)	65.1 (49.1–79.0)	< 0.001
Loss of clear zone	71.6 (60.0–81.5)	0.46	53.5 (37.7–68.8)	96.8 (8.3–99.9)	< 0.001
Placental bulging	66.2 (54.3–76.8)	0.38	100 (88.8–100)	41.9 (27.0–57.9)	< 0.001
Exophytic mass	66.2 (54.3–76.8)	0.38	100 (88.8–100)	41.9 (27.0–57.9)	< 0.001

*Except for new lacuna, all other indexes were evaluated in individuals considered high risk

†New lacuna was considered as grade 2 and 3 in sonography evaluation

**Fig. 2:**
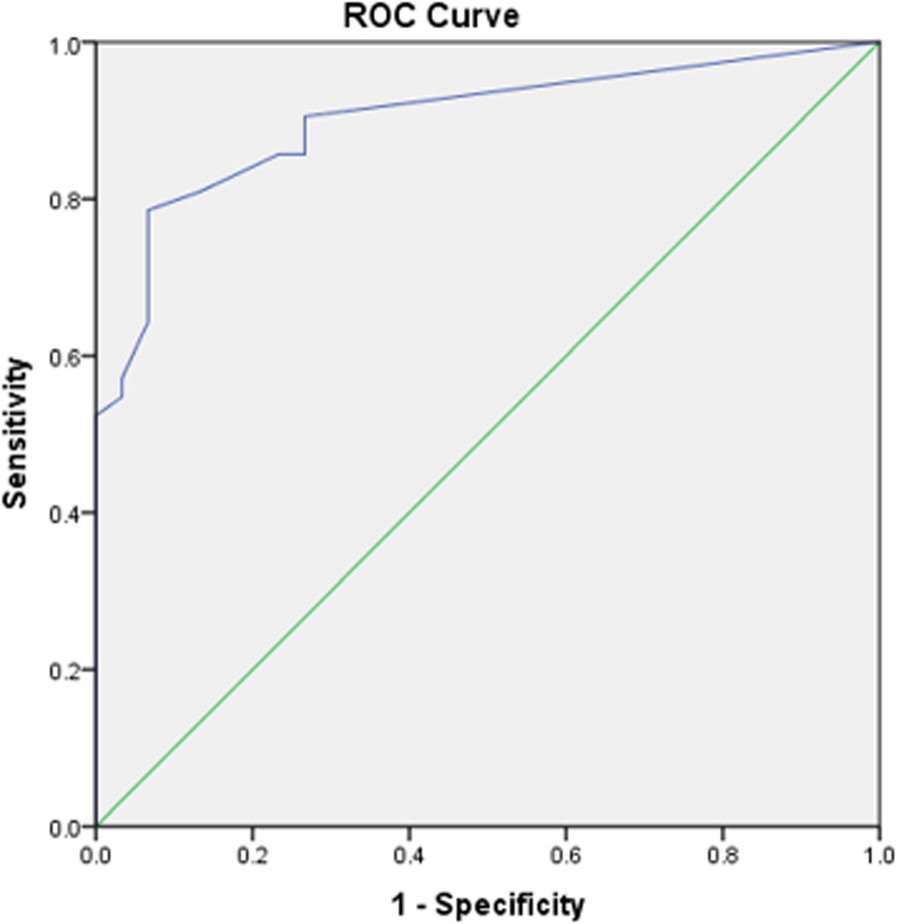
Receiver operator characteristic (ROC) curve analysis for the diagnosis of accreta based on lacuna flow rates

Using the ROC curve analysis we determined the optimum cut-off point based on flow in lacuna structures for the diagnosis of PAS. Accordingly, a cut-off of 11.5 cm/s was defined, which had a sensitivity of 79% and specificity of 93% and an accuracy of 90% for the diagnosis of PAS (Fig. 2).

**Fig. 3:**
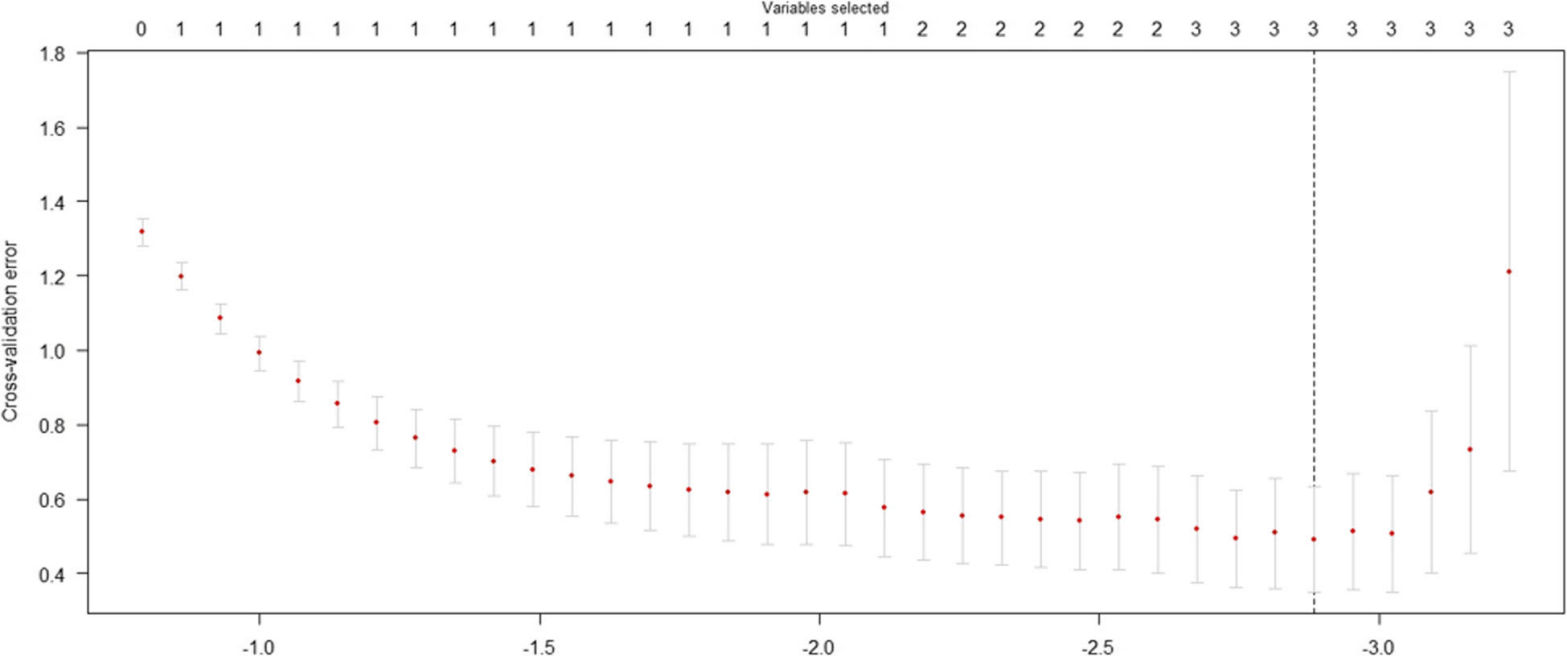
This shows the tuning parameter which was estimated as 0.011 using the 5-fold cross validation technique

In the regression analysis, the tuning parameter was estimated as 0.011 using the 5-fold cross validation technique (Fig. 3).

Imposing the tuning parameter on the MCP logistic regression, lead to the elimination of 12 out of 15 variables from the model. The proposed model revealed that utero-vesical hypervascularity, bladder interruption and new lacunae have significant contribution in PAS. In addition probability of PAS for each case was calculated using MCP logistic model as followed:

$$ \mathrm{pi}=\frac{e^{-10.4+5.78\ast \mathrm{Bladder}\ \mathrm{interuption}+7.84\ast \mathrm{utero}-\mathrm{vesicular}\ \mathrm{vascularity}+5.97\ast \mathrm{new}.\mathrm{lacunae}}}{1+{e}^{-10.4+5.78\ast \mathrm{Bladder}\ \mathrm{interuption}+7.84\ast \mathrm{utero}-\mathrm{vesicular}\ \mathrm{vascularity}+5.97\ast \mathrm{new}.\mathrm{lacunae}}} $$

Optimal cut off point was determined as *p* = 0.51 in the ROC analysis. Probability of PAS for women with lacunae was between 96 and 100% and probability of lacunae for women without PAS was between 0 to 7%, therefore accuracy of the proposed model was equal to 100%.

## Discussion

We aimed to introduce a model for the diagnosis of PAS based on sonography indexes in a group of high risk women for invasive placentation, furthermore we also compared each sonography index with the gold standard diagnosis of PAS and obtained the agreement and diagnostic value of each index, separately. We found that for the diagnosis of PAS all previously known sonography indices including: existence of abnormal placental lacuna, bladder interruption, myometrial thinning, placental bulging, existence of exophytic mass, utero-vesical vascularity, subplacental vascularity, bridging vessels, and loss of clear zone are significantly higher among those with PAS. Furthermore, we found that among all these indices utero-vesical vascularity, bridging vessels, bladder interruption, and subplacental vascularity have the highest agreement with gold standard diagnosis. As the main goal of our study we defined a model based on existing sonography indices to define the chances of having PAS which had a 100% accuracy. In our model the three indices of utero-vesical hypervascularity, bladder interruption and new lacunae were predictive of PAS. In addition, existence of abnormal lacuna structures alone had a predicting power of 96 to 100% for PAS. We also found that using lacuna flow rate at a cut-off of 11.5 cm/s can predict PAS with an accuracy of 90%.

Similar to that reported in our study, Comstock et al. [20] reported that visualization of placental lacuna to be the most reliable diagnostic sign [sensitivity: 93% and positive predictive value (PPV): 93%] for the diagnosis of accreta among women with a history of Cesarean section in their third trimester (for most of their patients) of pregnancy. Although their study included limited number of sonography criteria, their report was in concordance with our findings as we found that existence of lacuna structures in our final model could predict accreta by a precision 96 and 100% during the third trimester of pregnancy. Their findings also showed that existence of lacunae structures during 15–40 weeks of gestational age compared to 15–20 weeks of gestational age had a better sensitivity and PPV. Aside to the larger population size and the different analysis performed between the two studies, this study showed that existence of lacuna may be more reliable as gestational age increases and considering that in our study all patients were in their third trimester of pregnancy and most recent radiological images were considered for evaluation, this may be among the reasons why we had a significantly higher recorded accuracy for the lacunae index in detecting accreta. A narrative review by D’Antonio et al. [1] in 2016, reported that among 23 studies that evaluated placental lacuna diagnostic efficiency, a pooled sensitivity of 77%, specificity of 95%, PLR of 4.5%, NLR of 0.29%, and diagnostic odds ratio of 24.32% was reported for the diagnosis of invasive placentation.

D’Antonio [1] reported a pooled sensitivity of 49%, specificity of 99.75%, PLR of 30.56%, NLR of 0.51%, and diagnostic odds ratio of 93.7% for bladder interruption for the diagnosis of invasive placentation. We found this index to be significant in our final model for the prediction of PAS, furthermore this index had a high agreement score with that of the gold standard, which shows that in most occasions this index is measured in accordance with that of the gold standard modality. Regarding bladder interruption, Cumstock et al. [20] found a sensitivity of 20% and PPV of 75% for the detection of accreta. Bladder interruption was also seen among all patients with either accreta or increta in a small older series by Hoffman-Tretin and colleagues [21].

Utero-vascular hypervascularity was another factor that presented as significant in our model. In a recent study by Maged et al. [22] a total of 100 individuals with a history of Cesarean section were evaluated and compared in two groups, those with accreta and those without accreta. Similar to our study, they found all sonography indices in their study (loss of clear zone, lacunae, utero-vesical hypervascularity, and subplacental hypervascularity) to be significantly higher in the accreta group. They found utero-vesical hypervascularity to have a sensitivity of 47.6%, specificity of 94.5%, PPV of 93.7, and 51.4%, and accuracy of 65% for diagnosis of accreta. They also found lacunae structures to have a sensitivity of 93.6%, specificity of 62.1%, PPV of 80, 85% of NPV, and accuracy of 82%.

In a meta-analysis published in 2013 by Meng et al. [8], the diagnostic value of US and MRI were compared with regards to detecting placenta accreta. They found that among a total of 13 studies that compared the two diagnostic modalities, US had a sensitivity of 83% and specificity of 95% and MRI had a sensitivity of 82% and specificity of 88% for the diagnosis of accreta. They concluded that US and MRI do not differ regarding diagnostic value.

Although other diagnostic modalities such as MRI are done to confirm diagnosis of placenta accreta in posterior and some cases of lateral placental site implantation [1], our findings show that using the introduced model, almost 100% of all cases of PASs are diagnosed. If supported by future studies our model can significantly aid in the diagnosis of invasive placentation among high risk (considered those with a low lying placenta or previa) women with previous history of Cesarean sections.

To the best of the authors’ knowledge, this is the first model to predict PAS among women with previous Cesarean section with a probability of almost 100%, and among the most comprehensive studies that has included all sonography indices for prediction of PAS. If further supported by future studies, the model can be used to diagnose the existence of PAS among women with a history of Cesarean sections and low lying placenta or placenta previa using only US parameters.

This study was not without limitation. First is the nature of US, as it is operator dependent, all measurements need to be done by an expert in sonography. However, for the first time, we did use standardized definitions provided by the EW-AIP in our study in order to alleviate this issue [12]. Overall, due to the nature of our study and the rarity of PAS, similar to previous literature, we did not have a large sample of individuals with PAS, and accordingly our final model needs to be verified by future literature. However, we did use advanced statistical modeling to eliminate this issue. Among limitations of the study could be that, due to the high costs of pathology evaluation of the placenta, those who had spontaneous extraction of placenta during Cesarean section did not undergo pathology evaluation for focal accreta.

Among the limitation of the current report is that some forms of PAS including a less aggressive form of placenta accreta may be managed with conservative surgery, on the other hand more aggressive forms require a more radical approach. As our primary outcome was considered to be the existence of PAS we did not stratify patients based on the degree of PAS (placenta accreta/increta versus placenta percreta) and thus our model can only be used for the diagnosis of PAS and does not allow a classification of severity of the condition, which represent one of the main issues in the current management of PAS.

Another issue relates to the statistical modeling used in our study, MCP is among penalized methods, and although penalized methods were initially developed for high-dimensional conditions and data, in conditions where variables may be correlated, even when less than 10 variables exist, this method is an excellent choice. Furthermore, in the presence of multicollinearity, traditional variable selection techniques such as stepwise, forward and backward methods can lead to misleading results as they are aggressive methods. In short, penalized regression presence among the best choices even in low dimensional data [23]. In MCP, we include all existing variables (potential risk factors meaning all the variables in the current study) in the final model as in other penalized models, and perform simultaneous estimation and variable selection. Nature of the penalty function, which is added in the maximum likelihood of the model, forces some coefficients to shrink to zero and eliminates redundant variables from the model.

As recent findings have indicated that perhaps scar from previous Cesarean section may be among the primary causes of accreta [24], in here we only included individuals with a history of Cesarean sections and our final model is only applicable in these individuals.

Considering that at the time of our study the new FIGO guidelines did not exist for the definition of PAS, future studies would benefit from the use of more recent definition proposed by the “FIGO Safe Motherhood and Newborn Health Committee” and to focus on models that aside to the diagnosis of PAS, would provide a means for stratification of severity of PAS as well.

## Conclusions

In conclusion, we found that using the introduced model based on three factors of abnormal lacuna structures (grades 2 and 3), bladder wall interruption and utero-vesical vascularity, 100% of all cases of PASs are diagnosable among women with previous Cesarean sections.

## Abbreviation

MCP: Minimax Concave Penalty
PAS: Placenta accreta spectrum
PPV: Positive predictive value
ROC: Receiver operator characteristic
US: Ultrasonography

## References

[1] D'antonio F (2016). Counseling in fetal medicine: evidence-based answers to clinical questions on morbidly adherent placenta. Ultrasound Obstet Gynecol.

[2] Shellhaas CS (2009). The frequency and complication rates of hysterectomy accompanying cesarean delivery. Obstet Gynecol.

[3] Miller DA, Chollet JA, Goodwin TM (1997). Clinical risk factors for placenta previa-placenta accreta. Am J Obstet Gynecol.

[4] Balayla J, Bondarenko HD (2013). Placenta accreta and the risk of adverse maternal and neonatal outcomes. J Perinat Med.

[5] O'Brien JM, Barton JR, Donaldson ES (1996). The management of placenta percreta: conservative and operative strategies. Am J Obstet Gynecol.

[6] Silver RM (2006). Maternal morbidity associated with multiple repeat cesarean deliveries. Obstet Gynecol.

[7] Al-Khan A (2014). Maternal and fetal outcomes in placenta accreta after institution of team-managed care. Reprod Sci.

[8] Meng X, Xie L, Song W (2013). Comparing the diagnostic value of ultrasound and magnetic resonance imaging for placenta accreta: a systematic review and meta-analysis. Ultrasound Med Biol.

[9] Berkley EM, Abuhamad AZ (2013). Prenatal diagnosis of placenta accreta: is sonography all we need?. J Ultrasound Med.

[10] D'Antonio F (2014). Prenatal identification of invasive placentation using magnetic resonance imaging: systematic review and meta-analysis. Ultrasound Obstet Gynecol.

[11] Bowman ZS (2014). Interobserver variability of sonography for prediction of placenta accreta. J Ultrasound Med.

[12] Collins SL (2016). Proposal for standardized ultrasound descriptors of abnormally invasive placenta (AIP). Ultrasound Obstet Gynecol.

[13] Jauniaux E (2016). Accreta placentation: a systematic review of prenatal ultrasound imaging and grading of villous invasiveness. Am J Obstet Gynecol.

[14] Hamada S (2011). Ultrasonographic findings of placenta lacunae and a lack of a clear zone in cases with placenta previa and normal placenta. Prenat Diagn.

[15] Finberg HJ, Williams JW (1992). Placenta accreta: prospective sonographic diagnosis in patients with placenta previa and prior cesarean section. J Ultrasound Med.

[16] Shamshirsaz, A.A., et al, Maternal morbidity in patients with morbidly adherent placenta treated with and without a standardized multidisciplinary approach. Am J Obstet Gynecol, 2015. 212(2): p. 218. e1–218. e9. 10.1016/j.ajog.2014.08.019 25173187

[17] Dannheim K, Shainker SA, Hecht JL (2016). Hysterectomy for placenta accreta; methods for gross and microscopic pathology examination. Arch Gynecol Obstet.

[18] Calì G (2013). Morbidly adherent placenta: evaluation of ultrasound diagnostic criteria and differentiation of placenta accreta from percreta. Ultrasound Obstet Gynecol.

[19] Youden WJ (1950). Index for rating diagnostic tests. Cancer.

[20] Comstock CH (2004). Sonographic detection of placenta accreta in the second and third trimesters of pregnancy. Am J Obstet Gynecol.

[21] Hoffman-Tretin JC (1992). Placenta accreta. Additional sonographic observations. J Ultrasound Med.

[22] Maged AM, et al. Prevalence and diagnostic accuracy of Doppler ultrasound of placenta accreta in Egypt. J Matern Fetal Neonatal Med. 2017:1–7. 10.1080/14767058.2017.1303667 28264611

[23] Shmueli G (2010). To explain or to predict?. Stat Sci.

[24] Timor-Tritsch I (2014). Cesarean scar pregnancy and early placenta accreta share common histology. Ultrasound Obstet Gynecol.

